# Enhancing malignant transformation predictions in oral potentially malignant disorders: A novel machine learning framework using real-world data

**DOI:** 10.1016/j.isci.2025.112062

**Published:** 2025-02-18

**Authors:** Jing Wen Li, Meng Jing Zhang, Ya Fang Zhou, John Adeoye, Jing Ya Jane Pu, Peter Thomson, Colman Patrick McGrath, Dian Zhang, Li Wu Zheng

**Affiliations:** 1 Division of Oral & Maxillofacial Surgery, Faculty of Dentistry, The University of Hong Kong, Hong Kong SAR, China; 2 Department of Computer Science and Software Engineering, Shenzhen University, Shenzhen, China; 3 School of Medicine and Dentistry, Griffith University, Queensland, Australia; 4 Division of Applied Oral Sciences & Community Dental Care, Faculty of Dentistry, The University of Hong Kong, Hong Kong SAR, China

**Keywords:** Public health, Artificial intelligence

## Abstract

This study addresses the challenge of accurately predicting malignant transformation risk in patients with oral potentially malignant disorders (OPMDs). Using data from 1,094 patients across three institutions (2004–2023), the researchers compared traditional statistical methods, including a Cox proportional hazards (Cox-PH) nomogram, with machine learning (ML) algorithms. A novel Self Attention Artificial Neural Network (SANN) model was developed, trained, and validated alongside other ML models including ANN, RF, and DeepSurv. The SANN model outperformed all other approaches, achieving an AUC of 0.9877, with sensitivity, specificity, accuracy, and precision exceeding 0.96. In comparison, the Cox-PH nomogram achieved AUCs of 0.880–0.902. Comprehensive evaluations using Receiver Operating Characteristic, calibration curves, and decision curve analysis demonstrated SANN’s superior predictive efficacy, robustness, and generalizability. These findings highlight the potential of customized ML models, particularly SANN, to enhance early identification and management of high-risk OPMD patients, outperforming conventional statistical methods.

## Highlights

• The malignancy potential of OPMD highlights the importance of early detection and management
• Machine learning models outperform traditional statistics in predicting cancer progression
• The proposed SANN model provides accurate predictions, supporting clinical decision-making
• SANN shows strong generalizability and is well suited for clinical integration

## Graphical abstract

**Figure:**
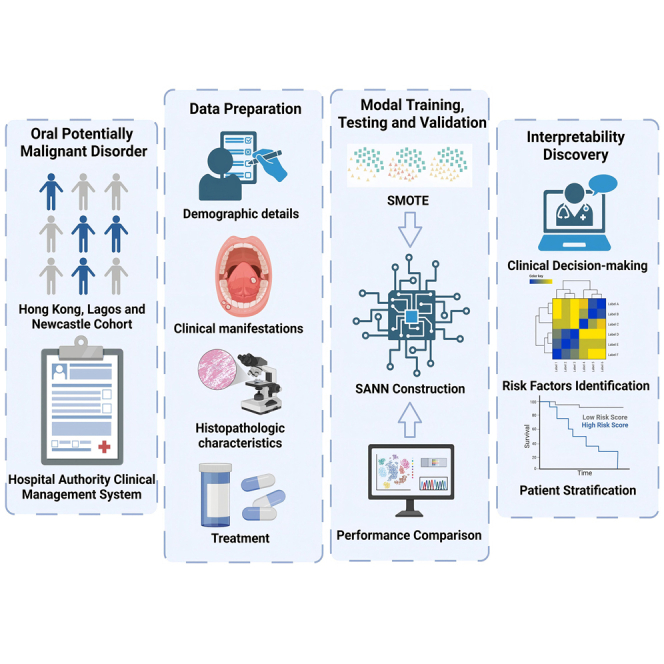

## Introduction

Oral squamous cell carcinoma (OSCC) represents a prevalent global malignancy, ranking as the 16^th^ most common cancer and resulting in over 177,000 fatalities annually.^1^ A decline has been reported in the 5-year survival rate of OSCC in correlation with disease progression, from 86.3% during the localized stage to 39.3% at the distant stage.^2^ Therefore, early prediction, detection, and diagnosis of OSCC are crucial in facilitating clinicians' ability to meticulously track disease progression and implement timely intervention and treatment strategies.

Oral carcinogenesis is characterized by an extended pre-pathologic phase, spanning the time between initial exposure to risk factors and the eventual onset of overt oral cancer. This pre-pathologic phase is often accompanied by the development of oral potentially malignant disorders (OPMDs), which exhibit an elevated risk of malignant transformation (MT).^3^ Among this collection of OPMDs, oral lichen planus (OLP) and oral lichenoid lesions (OLLs) are widely recognized as two of the more frequently encountered conditions in clinical practice. OLP is defined as a chronic inflammatory disorder with characteristic relapses and remissions, showing white reticular lesions with or without atrophic, erosive, and ulcerative and/or plaque-like areas,^4^ whereas OLL can be triggered by multiple results including allergic response to dental materials, usage of certain medications, graft-vs-host disease (GVHD) and systematic disease.^5^^,^^6^ The malignancy rates of OPMDs range from 2.6% to 7.9%^7^^,^^8^^,^^9^^,^^10^; for OLP and OLL, the rates vary from 0.4% to 12.5% and 1.2% to 4.4%, respectively.^11^^,^^12^^,^^13^ Numerous studies have identified a range of risk factors associated with the MT of OLP and OLL. These include patient characteristics such as age and gender, as well as clinical subtypes, systemic comorbidities, epithelial dysplasia, and lifestyle factors like tobacco smoking and alcohol consumption.^14^^,^^15^^,^^16^ However, currently, there exist limited clinical predictive models for MT of OPMDs; meanwhile, there remains substantial scope for improving the predictive efficacy of such models. Given the chronic, complex, multi-step nature of the progression toward malignancy, prediction for the MT potential of OPMDs should be based on extended longitudinal follow-up periods to maximize their real-world applicability.

Cox proportional hazards (Cox-PH) regression models are commonly utilized in cohort studies to identify risk factors and construct predictive models using survival data. Cox-PH can define the importance of variables based on hazard ratios, offering quantitative and interpretable results.^17^ Nomogram have become widely adopted in cancer management for predicting the risk of MT, leveraging the results from Cox-PH regression models, to provide visualized predictions.^18^ However, Cox-PH is limited by assumptions of proportional hazards and linearity, which, if violated, can impact the model’s predictive performance. Furthermore, Cox-PH models struggle to handle non-linear, complex relationships among variables.^19^^,^^20^ Nowadays, artificial intelligence (AI)-based techniques enable computers to learn from processed data and information, developing decision-making capabilities through iterative experience-based improvements.^21^^,^^22^ A large number of conventional machine learning (ML) and deep learning (DL) methods have been applied for survival analysis. These approaches offer the ability to reach non-linear classifications or do not require *a priori* selection of covariates, instead learning them adaptively.^23^^,^^24^ However, the most existed models currently proposed for MT have defined the outcome as a binary classification, rather than incorporating "real-world data" collected from actual clinical practice or generating outcomes as a probability of transformation over time.^25^^,^^26^ Real-world data reflect the true diversity of patient populations, treatments, and outcomes seen in everyday medical practice, rather than the more homogeneous populations and standardized protocols of clinical trials. In addition, the current repertoire of algorithms employed in survival analysis exhibits considerable untapped potential for advancements in the realms of feature selection and interpretability, particularly when confronted with high-dimensional datasets.^27^^,^^28^ The comparative effectiveness of traditional Cox-PH models and existing ML algorithms in predicting the risk of MT in OPMDs is an ongoing area of exploration. Meanwhile, developing a novel approach in MT prediction for OPMDs and enhancing the model’s capability of feature extraction are currently indispensable.

In this context, the aim of this study was 2-fold. First, it aimed to compare the predictive performance between Cox-PH regression models and ML algorithms. Second, we sought to propose a customized ML framework specifically designed to achieve accurate prediction of MT potential in OPMDs based on real-world data.

## Results

### Baseline characteristics and risk factors of the study population

**Figure 1:**
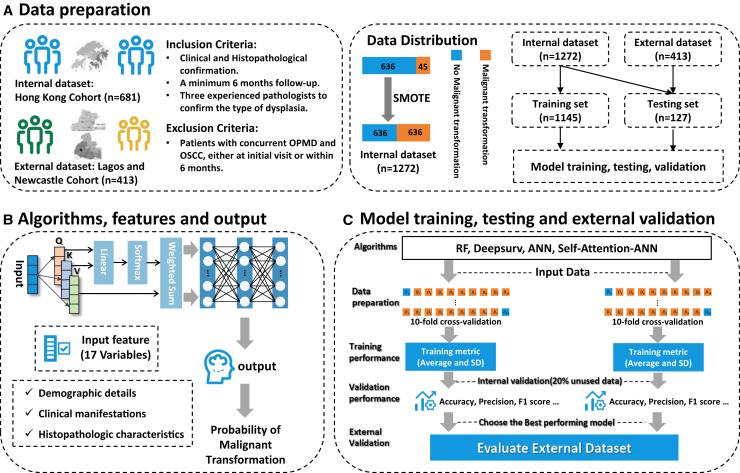
Overview of the development and validation of a machine-learning-based prediction system for OPMDs malignant transformations (A) Data collection and preprocessing. (B) Structure of self-attention ANN model. (C) Model training and evaluation.

This study encompassed a sample size of 681 OPMDs patients, comprising 444 cases of OLP and 237 cases of OLL. The rate of MT was found to be 6.608%, with the average follow-up duration of 8.62 years. Based on the 2003 World Health Organization (WHO) modified diagnostic criteria, the exclusion of cases with dysplasia at the initial diagnosis results in an overall reduction of the MT rate of the included patients to 3.185%. Females exhibited a higher likelihood of the disease, with the prevalence ratio being 1:2.1 (male to female), as well as the MT rate in females (7.3%) exceeded that of males (5.0%). OLP and OLL were notably prevalent among the elderly population, with 76.2% of patients being over 60 years old. The MT rate among individuals aged 60 years and above (8.3%, 43 cases) was much higher compared to those below 60 years old (1.2%, 2 cases). A total of 192 patients (28.2%) had a history of smoking and/or alcohol consumption or persisted in these habits following the diagnosis. Furthermore, over half of the patients developed OPMD concomitant with systemic diseases (388 cases, 57.0%), whereas 12.5% (85 cases) had a history of previous malignancy other than oral cancer. In regard to the clinical sub-type, the prevalence is comparable (white: 336 cases, 49.3%; red: 345 cases, 50.7%); however, the MT rate of red lesions (9.6%) is approximately three times higher than that of white sub-type (3.6%). Besides, the most comment lesion site was buccal mucosa (417 cases, 61.2%), followed by multiple sites (88 cases, 12.9%) and tongue (77 cases, 11.3%) (Figure 1). The baseline characteristics of the study population are shown in Table 1.

**Table 1:** Demographic and clinicopathological characteristics of study population

Characteristics	Total *n* = 681	Patients with MT N(%) = 45 (6.61)	Patients without MT N(%) = 636 (93.39)	*p* value
Gender				0.322
Male	218 (32.0)	11 (5.0)	207 (95.0)	
Female	463 (68.0)	34 (7.3)	429 (92.7)	
**Age**				**<** 0.001^a^
< 60	162 (23.8)	2 (1.2)	160 (98.8)	
≥60	519 (76.2)	43 (8.3)	476 (91.7)	
Smoker or Drinker				0.085
SD	192 (28.2)	18 (9.4)	174 (90.6)	
NSND	489 (71.8)	27 (5.5)	462 (94.5)	
Systemic Comorbidities				0.061
Yes	388 (57.0)	32 (8.2)	356 (91.8)	
No	293 (43.0)	13 (4.4)	280 (95.6)	
Hypertension				0.153
Yes	261 (38.3)	22 (8.4)	239 (91.6)	
No	420 (61.7)	23 (5.5)	397 (94.5)	
Diabetes Mellitus				0.854
Yes	155 (22.8)	11 (7.1)	144 (92.9)	
No	526 (77.2)	34 (6.5)	492 (93.5)	
Hepatitis virus infection				0.788
Yes	60 (8.8)	3 (5.0)	57 (95.0)	
No	621 (91.2)	42 (6.8)	579 (93.2)	
**GVHD**				0.017^a^
Yes	9 (1.3)	3 (33.3)	6 (66.7)	
No	672 (98.7)	42 (6.3)	630 (93.8)	
Previous Malignancy				0.248
Yes	85 (12.5)	8 (9.4)	77 (90.6)	
No	596 (87.5)	37 (6.2)	559 (93.8)	
**Type of Lesions**				0.051
OLP	444 (65.2)	23 (5.2)	421 (94.8)	
OLL	237 (34.8)	22 (9.3)	215 (90.7)	
**Clinical Sub-type**				0.002^a^
White	336 (49.3)	12 (3.6)	324 (96.4)	
Red	345 (50.7)	33 (9.6)	312 (90.4)	
**Lesion Site**				**<** 0.001^a^
Buccal mucosa	417 (61.2)	12 (2.9)	405 (97.1)	
Tongue	77 (11.3)	18 (23.4)	59 (76.6)	
Gingiva	50 (7.3)	1 (2.0)	49 (98.0)	
Others	49 (7.2)	4 (8.2)	45 (91.8)	
Multiple	88 (12.9)	10 (11.4)	78 (88.6)	
**Ulceration or Erosion**				0.008^a^
Yes	371 (54.5)	33 (8.9)	338 (91.1)	
No	310 (45.5)	12 (3.9)	298 (96.1)	
Treatment				0.495
Medical	463 (68.0)	28 (6.0)	435 (94.0)	
Excision	109 (16.0)	10 (9.2)	99 (90.8)	
No treatment	109 (16.0)	7 (6.4)	102 (93.6)	
**OED at First Diagnosis**				**<** 0.001^a^
Yes	29 (4.3)	8 (27.6)	21 (72.4)	
No	652 (95.7)	37 (5.7)	615 (94.3)	
**OED During Follow-up**				**<** 0.001^a^
Yes	27 (4.0)	19 (70.4)	8 (29.6)	
No	654 (96.0)	26 (4.0)	628 (96.0)	
**OED Grade**				**<** 0.001^a^
Mild	22 (3.2)	6 (27.3)	17 (72.7)	
Moderate	14 (2.1)	7 (50.0)	8 (50.0)	
Severe	17 (2.5)	12 (70.6)	6 (29.4)	
No dysplasia	628 (92.2)	20 (3.2)	608 (96.8)	

a *p* < 0.05 indicates statistical significance.

To further identify the risk factors for malignant progression of OPMDs, Cox-PH regression analysis was performed. Univariate Cox regression analysis identified 11 variables that were statistically significant risk factors as shown in Table 2. Multivariate Cox regression revealed that the risk of MT of OPMDs increased in the OLL (hazard ratio [HR] = 2.216, 95% CI = [1.155, 4.250]; *p* = 0.017) as well as red lesions (HR = 2.391, 95% CI = [1.213, 4.712]; *p* = 0.012]. The risk of MT for lesions on the tongue (95% CI = [2.155, 10.348]; *p* < 0.001) and multiple sites (95% CI = [1.184, 7.180]; *p* = 0.013) was 4.722 and 2.916 times higher than that of affecting the buccal mucosa, respectively. Furthermore, the presence of OED, whether at initial visit (HR = 4.210, 95% CI = [1.864, 9.511]; *p* < 0.001) or during subsequent follow-up (HR = 11.850, 95% CI = [6.114, 22.964]; *p* < 0.001), was found to be significantly associated with an increased likelihood of MT.

**Table 2:** Univariate & multivariate cox regression analyses and Bayesian Cox regression model of risk factors of malignant transformation of OLCs

Variable	Univariate Cox regression analysis		Multivariate Cox regression analysis		Bayesian Cox regression model
	HR (95% CI)	*p* value	HR (95% CI)	*p* value	HR (95% CI)
Gender					
Male	1.403 (0.710, 2.771)	0.329			
Female	Reference				
**Age**					
< 60	5.055 (1.221, 20.921)	0.025^a^			
≥60	Reference				
**Smoker or Drinker**					
SD	Reference				
NSND	1.926 (1.055, 3.515)	0.033^a^			
**Systemic Comorbidities**					
Yes	Reference				
No	1.942 (1.018, 3.705)	0.044^a^			
Hypertension					
Yes	Reference				
No	1.501 (0.834, 2.700)	0.176			
Diabetes Mellitus					
Yes	Reference				
No	1.058 (0.536, 2.088)	0.871			
Hepatitis Virus Infection					
Yes	0.605 (0.187, 1.958)	0.402			
No	Reference				
**GVHD**					
Yes	Reference				
No	7.400 (2.282, 23.991)	**<** 0.001^a^			
Previous Malignancy					
Yes	Reference				
No	1.895 (0.880, 4.081)	0.102			
**Type of Lesions**					
OLP	2.137 (1.187, 3.846)	0.011^a^	2.216 (1.155, 4.250)	0.017^a^	Reference
OLL	Reference		Reference		0.568 (0.353, 0.891)
**Clinical Sub-type**					
White	2.516 (1.296, 4.888)	0.006^a^	2.391 (1.213, 4.712)	0.012^a^	Reference
Red	Reference		Reference		0.424 (0.282, 0.648)
**Lesion Site**		< 0.001^a^		0.002^a^	
Buccal mucosa	Reference		Reference		Reference
Tongue	10.090 (4.821, 21.117)	**<** 0.001^a^	4.722 (2.155, 10.348)	**<** 0.001^a^	1.729 (1.006, 2.981)
Gingiva	0.933 (0.121, 7.196)	0.947	2.712 (0.940, 7.829)	0.730	
Others	2.948 (0.949, 9.154)	0.061	2.006 (0.635, 6.336)	0.236	
Multiple	4.641 (2.001, 10.761)	< 0.001^a^	2.916 (1.184, 7.180)	0.013	
**Ulceration or Erosion**					
Yes	Reference				
No	2.117 (1.090, 4.112)	0.027^a^			
Treatment		0.342			
No treatment	Reference				
Medical	0.982 (0.425, 2.274)	0.967			
Excision	1.682 (0.640, 4.422)	0.292			
**OED at First Diagnosis**					
Yes	Reference		Reference		Reference
No	7.798 (3.590, 16.941)	**<** 0.001^a^	4.210 (1.864, 9.511)	**<** 0.001^a^	2.497 (1.162, 4.899)
**OED during Follow-up**					
Yes	Reference		Reference		Reference
No	17.587 (9.653, 32.043)	**<** 0.001^a^	11.850 (6.114, 22.964)	**<** 0.001^a^	6.526 (3.711, 10.854)
**OED Grade**		**<** 0.001^a^			
No dysplasia	Reference				
Mild	13.252 (5.285, 33.226)	**<** 0.001^a^			
Moderate	16.302 (6.839, 38.858)	**<** 0.001^a^			
Severe	23.231 (11.270, 47.886)	**<** 0.001^a^			

a *p* < 0.05 indicates statistical significance; CI, confidence interval.

### Nomogram construction and validation

**Figure 2:**
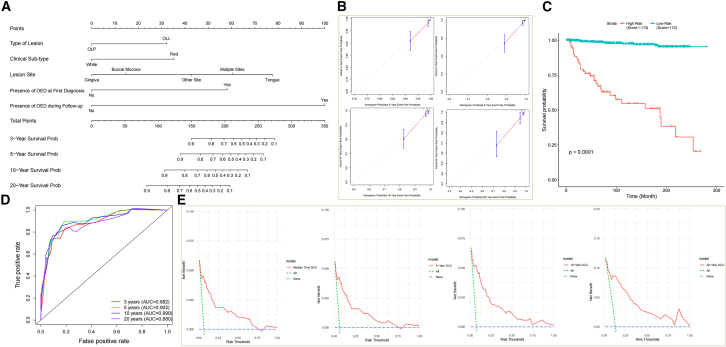
Evaluation of the nomogram prediction model for cancer risk assessment (A) Nomogram prediction model. The nomogram was used to calculate a total score for each patient by adding the scores obtained from individual characteristics. (B) Calibration curve with bootstrap (1,000 resample) validation showing the predicted line overlapped well with the reference line both in 3-year, 5-year, 10-year, and 20-year follow-up periods. (C) Kaplan-Meier survival curve. (D) Time-dependent receiver operating characteristic (ROC) curve analysis. (E) Decision curve analysis (DCA, median time, 5-year, 10-year, and 20-year) showing that the nomogram offered clinical relevance.

The nomogram was utilized to calculate a comprehensive score for individual by summing up the scores obtained from each risk factors. Based on the aforementioned analysis, the final nomogram incorporated the following parameters: lesion type, clinical sub-type, lesion site, and the presence of OED at initial diagnosis and during follow-up. By employing the calculated scores, the probabilities of event-free survival rates for various time periods up to 20 years were identified (Figure 2A). The generated nomogram for MT prediction exhibited a c-index of 0.8835, indicating a good predictive accuracy. Furthermore, calibration plots demonstrated that the nomogram was effectively calibrated, with the predicted probabilities aligning well with the actual probabilities for event-free possibilities at 3, 5, 10, and 20 years in these cohorts (Figure 2B). These findings indicate that the developed nomogram exhibited sufficient predictive capability within database. Moreover, a Bayesian Cox regression analysis was also performed to cross-validate the selected factors. The convergence of the Bayesian Cox regression model was assessed through trace plots of the iterative history. The 95% CIs of the hazard ratios for the various factors did not contain the value of 1, indicating statistically significant differences. The results indicated that all the variables incorporated into the nomogram demonstrated statistical significance within the Bayesian framework (Table 2).

In addition to the individualized predictions derived from the nomogram, we further categorized the patients into two groups based on their nomogram scores using a cutoff value. The cutoff value was determined by establishing a threshold of 95.8%, with patients scoring below 113 assigned to the low-risk group and those scoring higher than 113 assigned to the high-risk group. Statistical analysis using the log rank test revealed a significant difference between the Kaplan-Meier survival curves of the two groups, further confirming the prognostic value of the nomogram (p = 2e-16; Figure 2C). The sensitivity and specificity of the nomogram were assessed using time-dependent receiver operating characteristic (ROC) curve analyses. The area under the curve (AUC) for the model was 0.882 at the 3-year follow-up, 0.902 at the 5-year follow-up, 0.899 at the 10-year follow-up, and 0.880 at the 20-year follow-up. These AUC values indicate that the model demonstrated a good level of predictive performance across the examined time horizons (Figure 2D). Decision curve analysis (DCA) was conducted to evaluate the net benefit of a predictive model. The results demonstrated that nomogram exhibited clinical utility over a span of 20 years, underscoring its practical value in aiding clinical decision-making and patient management across a substantial time frame (Figure 2E).

### Machine learning model performance

**Figure 3:**
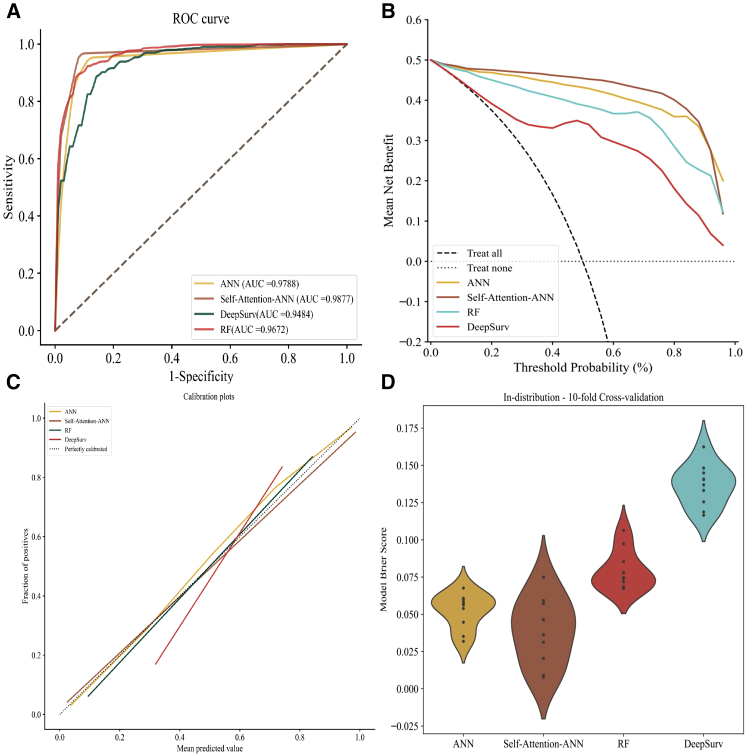
Performance evaluation of machine learning models for cancer risk assessment (A) Receiver operating characteristic (ROC) curve analysis. The area under the curve (AUC) for the model was 0.9877 (SANN), 0.9788 (ANN), 0.9672 (RF), and 0.9484 (DeepSurv). (B) Decision curve analysis showing that four models offered clinical relevance. (C) Calibration curve validation showing the predicted line overlapped well with the reference line in SANN, ANN, and RF. (D) Violin plots of the 10-fold cross-validation results for the Brier Score in four models.

The predictive performance metrics for the four machine learning models were presented in Table 3. The AUC for the four ML models uniformly exceeded 94%, and their sensitivity and specificity were generally higher than 90%, demonstrating an overall superior predictive performance compared to the nomogram prediction. Within this evaluation, SANN achieved the best AUC of 0.9877, closely followed by the ANN algorithm with an AUC of 0.9788 (Figure 3A). The cutoff values identified through ROC analysis for the five machine learning models were as follows: SANN (cutoff = 60.13%), ANN (cutoff = 44.25%), RF (cutoff = 61.84%), and DeepSurv (cutoff = 52.86%). At these respective cutoff thresholds, the DCAs for all models were observed to be situated above the None and All reference lines, with the SANN models exhibiting the most prominent clinical utility within this set (Figure 3B). In regard to the calibration curves, DeepSurv model exhibited the poorest alignment with the perfectly calibrated reference line, indicating suboptimal calibration performance. In contrast, the remaining three algorithms demonstrated relatively robust calibration, with their calibration curves closely tracking the ideal perfectly calibrated line across the range of predicted survival probabilities (Figure 3C). Comprehensive evaluation of the ROC, AUC, calibration curves, and DCA across the four algorithms revealed that the SANN model exhibited the best predictive efficacy and stability, followed by ANN algorithm. In contrast, the DeepSurv model demonstrated poorer consistency between its predicted probabilities and the actual observed outcomes.

**Table 3:** Performance of machine learning algorithms on the training, testing, and external validation datasets

Training & testing (external validation)									
	AUC	C-Index	Sensitivity	Specificity	Accuracy	Precision	Negative Predictive Value	F1-Score	Youden’s J Statistic
ANN	0.9788 (0.9554)	0.7564 (0.8750)	0.9497 (0.9000)	0.9388 (0.9233)	0.9442 (0.9184)	0.9405 (0.7898)	0.9497 (0.9729)	0.9446 (0.8313)	0.8885 (0.8233)
SANN	0.9877 (0.9617)	0.7643 (0.9078)	0.9656 (0.9250)	0.9593 (0.9467)	0.9623 (0.9421)	0.9603 (0.8524)	0.9665 (0.9799)	0.9623 (0.8779)	0.9249 (0.8715)
RF	0.9672 (0.9460)	0.9177 (0.9253)	0.9137 (0.9375)	0.9215 (0.8667)	0.9175 (0.8816)	0.9236 (0.7143)	0.9159 (0.9836)	0.9173 (0.7884)	0.8352 (0.8041)
DeepSurv	0.9484 (0.9379)	0.9478 (0.8812)	0.9292 (0.9374)	0.8823 (0.8967)	0.9057 (0.9053)	0.8905 (0.7316)	0.9290 (0.9831)	0.9077 (0.8123)	0.8115 (0.8341)

To validate the best-performing algorithms in terms of model discrimination and calibration, four algorithms were subjected to external validation using the Newcastle OPMD cohort for further evaluation. On the external validation dataset, the overall performance metrics of the models exhibited a slight decline relative to the testing cohort. Under this circumstance, SANN model continued to demonstrate superior performance, providing strong evidence of its external generalizability.

## Discussion

Accurate prediction of the malignant potential of OPMDs is crucial for the prevention and early diagnosis of oral cancer.^32^ Currently, there is a paucity of reliable decision-support models to aid clinicians in the rational assessment of MT risk and the formulation of appropriate patient follow-up and long-term monitoring strategies. As AI is increasingly being applied to precision medicine and outcome prediction, this study presents a comparative evaluation and validation of conventional Cox-PH and machine learning algorithms for predicting MT-free survival in patients with OPMDs. The results showed that ML algorithms demonstrated superior overall predictive performance compared to the Cox-PH model. The AUC for all selected ML models uniformly exceeded 94%, and their sensitivity and specificity were generally higher than 90%, indicting an overall superior performance compared to the nomogram prediction (AUC around 0.9). Specifically, the customized, enhanced model proposed in this work, referred to as SANN, achieved impressive performance metrics, including the AUC of 0.9877, sensitivity of 0.9656, specificity of 0.9593, accuracy of 0.9623, and precision of 0.9603. These results demonstrate robust discriminative capability and provided well-calibrated probability estimates as a function of time for the MT of OPMDs in both internal and external validation datasets.

**Figure 4:**
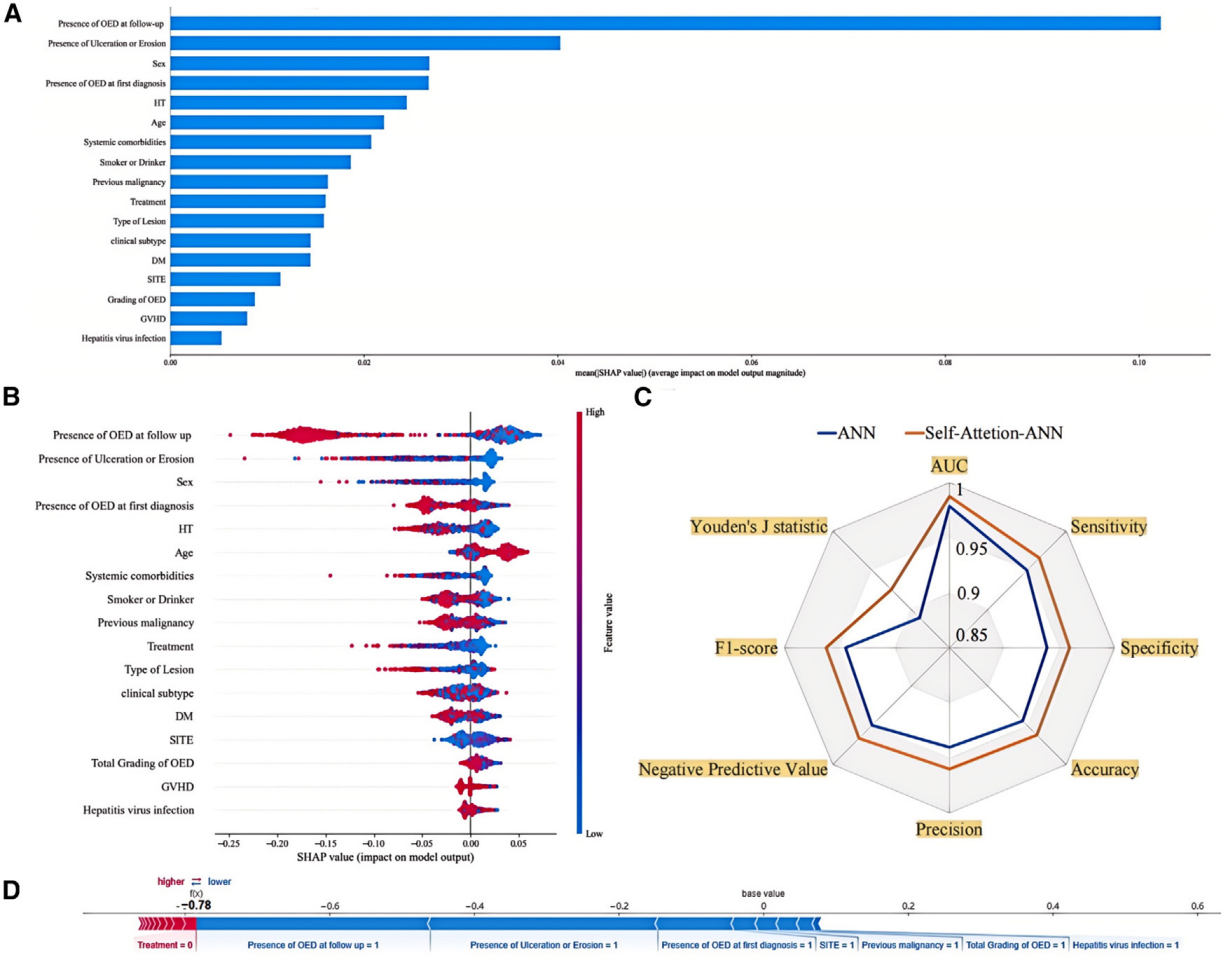
Interpretability of SANN model (A) The bar chart shows the shape of the self-attention weights for each input feature. The height of the bars represents the relative importance or "shape value" of each feature in contributing to the model’s output. Features with higher shape values indicate that the self-attention mechanism has placed greater emphasis on those inputs when making predictions. (B) The distribution of the effects of each feature on the model’s output. The color of the dots indicates the feature values, with red indicating higher values and blue representing lower values. (C) The spider plot illustrates the comparative performance of ANN and the SA-ANN model across multiple evaluation metrics. (D) The red and blue bars indicate risk factors and protective factors, respectively; longer bars signify greater importance of the corresponding features.

ANNs are composed of interconnected units, known as neurons, organized into different layers. The connections between these neurons are weighted, forming the edges of the network. The first layer of an ANN is responsible for receiving the input data, which then gets processed and propagated through multiple subsequent layers. This iterative process ultimately reaches the final output layer, allowing the network to generate predictions. This multilayered structure enables ANN to effectively handle tabular data and learn complex non-linear relationships within the input-output mapping.^33^ However, pure ANN models can struggle to adequately emphasize important information and capture long-distance data relationships. Rabadan et al. explored ANN-based cancer prognosis prediction models and noted that although ANN can effectively learn complex input-output mappings, they still face limitations such as difficulty in interpreting feature importance.^34^ The review by Kourou et al. also stated that although ANNs have shown excellent performance in cancer prediction, further improvements are also required, such as feature selection and data scarcity.^35^ To address this limitation, we explored the integration of self-attention mechanisms into the ANN framework that allows the model to dynamically focus on and weigh the most relevant features during the feature extraction process.^36^ By incorporating self-attention, the enhanced ANN model can better extract and utilize the crucial information in the data, potentially improving its performance in survival analysis tasks. According to the SHAP summary plots, which provide insights into the relative importance of different features in the model’s predictions, factors such as the presence of OED at first diagnosis or during follow-up, presence of ulceration or erosion, and the gender of patients demonstrate the strong impact on the model output, as indicated by their larger SHAP values. This clear representation allows for a better understanding of how each feature contributes to the predictive performance of the model (Figures 4A, 4B, and 4D). Our study found that the model architecture integrating self-attention exhibited comprehensive improvements in predictive performance metrics compared to traditional ANN models. Notably, SANN demonstrated marked improvements in the F1-score (ANN: 0.9446, SANN: 0.9623) and Youden’s J statistic (ANN: 0.8885, SANN: 0.9249) compared to the baseline ANN. The F1 score, which considers both the precision and recall of the model, highlights the enhanced accuracy and completeness of the SANN’s predictions. Youden’s J statistic, on the other hand, captures the model’s overall performance in discriminating between positive and negative samples. The simultaneous improvements in AUC, Accuracy, Sensitivity, Specificity, F1-score, and other related metrics reflect the comprehensive enhancement of the SANN model’s predictive capability for the malignant transformation of OPMDs, indicating an improved ability to better capture the complex relationships between the features and the model output, which leads to more accurate predictions (Figure 4C). Additionally, both nomogram and interpretability analysis of SANN model highlighted the significant contribution of dysplasia in the risk of MT (Figures 2A, 4A, 4B, and 4D). These findings underscore the critical role of dysplasia as an indicator of malignant potential for OPMD patients. Whether dysplasia is present at initial diagnosis or emerges during follow-up, it should be regarded as a crucial marker that warrants heightened clinical attention and management. The enhanced predictive capability of the SANN model, coupled with the insights derived from the feature importance analysis, provide compelling evidence to guide more vigilant monitoring and appropriate interventions for OPMD patients with OED lesions.

Moreover, it is worth noting that part of previous studies have overlooked a critical methodological issue—the lack of adherence to the principle of “sampling balance” during data training phase. This oversight can lead to the development of models that are biased toward the majority class, compromising their ability to accurately predict the minority class, which is often the clinically relevant outcome. To address this methodological limitation, we performed SMOTE preprocessing on the data to ensure a balanced representation of positive and negative cases.^37^ This approach guarantees that the reported performance metrics accurately reflect the true efficacy of the models in predicting the MT of OPMDs, fundamentally safeguarding the reliability of the study’s findings.

In summary, the malignant potential inherent to OPMDs should be duly emphasized, as early detection and appropriate intervention represent the fundamental cornerstones for the prevention of cancer progression. This study has demonstrated that machine-learning-based predictive models exhibit satisfactory levels of sensitivity, specificity, and accuracy in identifying OPMD patients at risk of MT, outperforming traditional nomogram-based approaches. Specifically, the customized SANN algorithm developed as part of this work has shown robust predictive capabilities to date. Furthermore, this research provides proof-of-concept that demographic, clinicopathological, and treatment data routinely captured in electronic health records can be effectively leveraged to forecast the risk of malignant transformation in OPMD cases. We encourage the utilization of multi-center real-world data for further model validation, as this could establish the developed tool as a reliable asset to inform critical clinical decision-making.

### Limitations of the study

To the best of our knowledge, this is the first and most comprehensive comparative study focused on both Cox-PH model and ML algorithms for OPMDs malignancy prediction. The study follows the modified 2003 WHO diagnostic criteria as the benchmark, allowing for the longest follow-up duration achieved thus far, and demonstrates long-term and currently the most accurate predictive capabilities. The proposed SANN model has achieved the most favorable performance metrics to date, thereby providing crucial clinical decision-making for assessing the MT risk of OPMDs to achieve the precision medicine. Despite the limited sample size, the extracted features have been formulated to be as comprehensive and detailed as practicable, which is particularly challenging to achieve in the context of retrospective studies. Notwithstanding the advancements in AI research across various medical domains, studies investigating the application of AI for the detection/prediction/prognosis of oral precancerous and cancerous lesions remain relatively scarce. Our experience from the current study calls for a more in-depth exploration and investigation of this specific area. Given these circumstances, the following clinical recommendations and considerations warrant attention.

(1) Future research should focus on multi-center real-world data to better capture regional differences and diversity. This will help improve the broad applicability and external validity of the research findings.
(2) Data collection methods should be more comprehensive and standardized. Retrospective analysis are often limited by the availability of data and the lack of unified data definitions, such as information on smoking and alcohol consumption frequency. Future real-world data collection should be more systematically standardized to improve model training and performance evaluation.
(3) The collection of samples often excludes complicated cases, and few studies take into account the general patient conditions. As a result, the reported diagnostic performance in the training and validation phases may not fully represent the effectiveness when applied in clinical practice.
(4) Uncertainty research regarding intelligent diagnosis of oral precancerous and cancerous has been limited. It is crucial to address the potential for erroneous judgments even with high confidence levels in the commonly used ML algorithms. AI-based diagnosis should provide explicit indications of its uncertainty or lack of knowledge to healthcare professionals and patients.
(5) Current research has primarily focused on textual data or image data (clinical photos or histopathological slides). Future studies should explore the integration of text and image data, aligning the lesion regions in the images with the corresponding textual information. This combined approach, coupled with advanced deep learning models, can provide more real-time and accurate predictions.
(6) From a public health perspective, disease prediction should not only consider clinical data but also account for "structural determinants"—the broad social factors that influence health and health inequalities. The focus should shift from a solely medical model to a more holistic and systematic approach to population health improvement, which can fundamentally control cancer progression and extend life expectancy.

## Resource availability

### Lead contact

Further information and requests for resources should be directed to the lead contact, Li Wu Zheng (lwzheng@hku.hk).

### Materials availability

This study did not generate any new materials.

### Data and code availability

• The data cannot be made publicly accessible due to hospital regulations. Distributing these data without the necessary consent could potentially breach patient confidentiality and contravene the approval granted by the Institutional Review Board for this study. Any additional information required to reanalyze the data reported in this paper is available from the lead contact on request.
• All original code has been deposited at https://github.com/zmj-szu/SANN and is publicly available as of the date of publication.
• Any additional information required to reanalyze the data reported in this paper is available from the lead contact upon request

## Acknowledgments

This study was supported by Stable Support Project of Shenzhen (No. 20231122145548001) and 10.13039/100016804Natural Science Foundation of Shenzhen Municipality (No. JCYJ20220531091407016).

## Author contributions

Concept and design, L.Z. and D.Z.; acquisition, analysis, or interpretation of data, L.Z., D.Z., P.T., J.L., M.Z., J.A., and J.J.P.; drafting of the manuscript, J.L. and M.Z.; critical revision of the manuscript for important intellectual content, all authors; statistical analysis, J.L., M.Z., and Y.Z.; obtained funding, D.Z.; administrative, technical, or material support, C.McG., Y.Z., P.T., J.A., and J.J.P.; supervision, L.Z. and D.Z.

## Declaration of interests

The authors declare no conflict of interest.

## STAR★Methods

### Key resources table

**Table:** 

REAGENT or RESOURCE	SOURCE	IDENTIFIER
**Deposited data**		

Raw Electronic Medical Record data	This paper	N/A
Code for model and network training and analysis	This paper	https://github.com/zmj-szu/SANN

**Software and algorithms**		

R version 4.3.3	R CRAN	https://cran.r-project.org/
Nomogram	R CRAN	https://cran.r-project.org/web/packages/rms/
Receiver Operating Characteristic	R CRAN	https://cran.r-project.org/web/packages/parTimeROC
Calibration curve analysis	R CRAN	https://cran.r-project.org/web/packages/pmcalibration
Decision curve analysis	R CRAN	https://cran.r-project.org/web/packages/dcurves
Python version 3.8	Python Software	https://www.python.org/
SHAP version 0.45.1	SHAP Software	https://shap.readthedocs.io/en/latest/
PyTorch version 2.1.2	PyTorch Software	https://pytorch.org/
Math	Python Software	https://docs.python.org/3/library/math.html
Copy	Python Software	https://docs.python.org/3/library/copy.html
OS	Python Software	https://docs.python.org/3/library/os.html
NumPy version 1.26.2	NumPy Software	https://numpy.org/
Pandas version 2.2.1	Pandas Software	https://pandas.pydata.org/
Scikit-learn version 1.4.1	Scikit-learn Software	https://scikit-learn.org/stable/api/sklearn.html
Lifelines version 0.28.0	Lifelines Software	https://lifelines.readthedocs.io/en/latest/
Matplotlib version 3.8.2	Matplotlib Software	https://matplotlib.org/stable/
Pytorchtools	TorchVision Package	https://github.com/Bjarten/early-stopping-pytorch
Imbalanced-learn version 0.12.2	Imbalanced-learn Software	https://imbalanced-learn.org/stable/
Openpyxl version 3.1.2	Openpyxl Software	https://openpyxl.readthedocs.io/en/stable/

### Experimental model and study participant details

The retrospective analysis included patients who were clinically and histopathologically diagnosed with OLP or OLL. Data spanning from 1st January 2004 to 31st December 2023 were collected from the Hong Kong Hospital Authority Clinical Management System (HA-CMS) of Queen Mary Hospital and Prince Philip Dental Hospital, Hong Kong. The diagnostic criteria for OLP and OLL were primarily adhered to the 2003 modified WHO criteria,^29^ with the exception that the presence of dysplasia was not regarded as an exclusion criterion. In accordance with the latest recommendations provided by The Working Group for whom Collaborating Centre for Oral Cancer in 2020,^30^ the presence of dysplasia resulted in a diagnosis of OLP with dysplasia or oral epithelial dysplasia with lichenoid features, and the patient was allocated to the OLP or OLL group accordingly.

Inclusion criteria were as follows: (a) Both clinical examination and histopathological observations were utilized to confirm the presence of OLP and OLL in all patients. (b) A minimum follow-up duration of 6 months was ensured for all patients. (c) Pathological review was conducted by three experienced pathologists to confirm the specific type of dysplasia present in each tissue sample; With regard to the exclusion criteria, patients who were diagnosed OLP or OLL along with OSCC during their initial visit or within 6 months after the first visit, were excluded from the study.

This study was conducted in accordance with the principles outlined in the Declaration of Helsinki. Approval to conduct the study was obtained from the Institutional Review Board of the University of Hong Kong/Hospital Authority Hong Kong West Cluster (Reference number: UW23-094). To ensure patient privacy and confidentiality, all clinical data were anonymized by the researchers, and any potential patient identifiers were removed prior to data analysis.

### Method details

#### Data extraction and preprocessing

The following data were collected for each case: (1) Patient status (alive or dead, cause of death), personal characteristics (age and gender, smoking and alcohol consumption); (2) Systematic disease (Diabetes Mellitus, Hypertension, Graft versus Host Disease, Hepatitis virus infection, Type of previous malignancy, Family history of malignancy); (3) Type of Lesions (OLP or OLL) and follow-up duration; (4) Clincial manifestations (clinical subtype, lesion site and size, presence of ulceration or erosion); (5) Presence of oral epithelial dysplasia at first diagnosis or during follow-up, grading of OED; (6) Treatment option; (7) Malignant transformation (Time-to-event, TNM stage, histologic variant of OSCC, prognosis). As for the risk-habit categories of smoking and alcohol consumption, we create a novel feature that classified patients into two groups: non-smoking, non-alcohol-drinking (NSND) patients and smoking and alcohol-drinking (SD) patients.

The feature selection process was applied to the dataset which involved removing variables with missing values that accounted for half the dataset, as well as eliminating redundant information. Meanwhile, features with a small number of missing values were imputed, ensuring no remaining missing data. One-hot encoding was used to encode the features, followed by feature value standardization. Furthermore, SMOTE (Synthetic Minority Over-sampling Technique) algorithm was employed to synthesize minority class samples, thereby achieving a balanced dataset between malignant and no-malignant cases while preserving the data distribution characteristics.

#### Algorithms selection and design

We proposed a customized, enhanced ML model specifically for the prediction of OPMD malignant transformation, named Self-attention Artificial Neural Network (SANN). ANN has shown unique advantages, such as non-linear modeling capability, automatic feature extraction, parallel computing, and improved interpretability, which have contributed to its competitive edge and promising application potential in the field of survival analysis. However, pure ANN models can struggle to adequately emphasize important information and capture long-distance data relationships. To enhance the feature extraction capabilities of the ANN model, we incorporate self-attention mechanisms. This framework reduces reliance on external information and effectively captures the inherent correlations among data or features, particularly excelling at modeling long-range dependencies. From the perspective of cancer risk prediction, different factors carry varying weights, which is crucial for accurate modeling. The SANN receives data at the input layer and employs self-attention to improve feature representation, enabling the model to better identify significant information related to cancer risk. The data is then transformed through multiple hidden layers, with the final outcome computed at the output layer. By leveraging self-attention, SANN accurately captures interdependencies between risk factors, allowing it to assign appropriate weights to each factor based on their relevance to cancer risk prediction, thereby enhancing the precision of feature extraction and modeling complex underlying relationships. The core formula underpinning the self-attention mechanism can be expressed as follows:

$$\text{Attention}\left(Q,K,V\right)=\text{softmax}\left(\frac{QK^{T}}{\sqrt{d_{k}}}\right)V$$

The scaling factor represents the magnitude of the dot product between the query (Q) and the corresponding key (K), where Q denotes the query input and K denotes the key input. Additionally, V represents the value input associated with the key-value pair. Detailed explanation of model principles was described in Data S1(1). Besides, three classical algorithms were selected to comparative evaluate their performance, based on their demonstrated effectiveness in previous survival analysis studies. These algorithms encompass a diverse range of methodological approaches, including regression models, deep learning, tree-based models, and specialized survival analysis techniques, namely Artificial Neural Network (ANN), Random Survival Forest (RSF), and DeepSurv.

#### Model training, testing and validation

Description of model construction and deployment phases underwent in this study was shown in Figure 1. To rigorously evaluate the performance of the aforementioned models, a 10-fold cross-validation procedure was performed. The datasets was randomly partitioned into 10 equal-sized subsets or folds, and then 10 iterations of training and testing were conducted, where in each iteration, 9 of the folds were used as the training set and the remaining fold was held out as the test set. The mean and standard deviation of the performance metrics were evaluated at the end of 10 iterations. Hyperparameters for the algorithms, such as learning rate, number of hidden layers, nodes per layer, dropout, and batch size, were tuned based on the performance measures at the algorithm level. The specific hyperparameter values considered are presented in Data S1(2). The model hyperparameters were tuned using only the training folds, and the model’s generalization capability was evaluated on the held-out test folds.

Furthermore, to validate the best-performing model(s), this study utilized a previously published dataset of 413 patients with OPMDs treated at the Maxillofacial Surgery Unit of the Newcastle Dental Hospital and the Royal Victoria Infirmary between August 1996 and December 2014. As the features available in this external datasets differed from those in the original training and internal validation data, we re-trained and re-validated the models using only the features available in the external datasets before conducting the final external validation.

### Quantification and statistical analysis

All conventional Cox regression analyses were performed using IBM SPSS Statistics 29.0.2.0. Univariate Cox regression analyses were employed to identify the risk factors associated with the malignant transformation of OPMDs, while multivariate Cox regression analyses were utilized to account for potential confounding factors. The backward selection stepwise regression method was applied to validate the inclusion of variables in the multivariate Cox regression model, ensuring that each independent variable in the model demonstrated statistical significance. The nomogram prediction model was developed using the results obtained from the regression analyses. To validate the nomogram, 1000 bootstrap resamples were employed to estimate the Harrell concordance index (C-index) values. These values were used to assess the discriminative ability of the nomogram in distinguishing patients who experienced malignant transformation from those who did not. The C-index, ranging from 0.5 to 1.0, serves as a measure of predictive discrimination, where a value of 0.5 indicates random chance and a value of 1 signifies perfect discrimination by the model.^31^ To accomplish this, R version 4.3.3 (http://www.R-project.org) was employed for conducting stepwise regression analysis, constructing the nomogram prediction model, performing Receiver Operating Characteristic (ROC) curve analysis, calibration curve analysis, and conducting decision curve analysis (DCA). Statistical significance was defined as P < 0.05.

In regards to the ML model performance, a comprehensive set of metrics including the C-index, Brier score loss, sensitivity, specificity, accuracy, precision, negative predictive value, F1 scores, and Youden’s J statistic were evaluated. Mathematical calculations for the performance metrics are given below:

$$\text{Sensitivity}=\frac{\text{TP}}{\text{TP}+\text{FN}}\;\text{Specificity}=\frac{\text{TN}}{\text{TN}+\text{FP}}$$

$$\text{Accuracy}=\frac{\text{TP}+\text{TN}}{\text{TP}+\text{FN}+\text{TN}+\text{FP}}\;\text{Precision}=\frac{\text{TP}}{\text{TP}+\text{FP}}$$

$$\text{Negative}\;\text{Predictive}\;\text{Value}=\frac{\text{TN}}{\text{TN}+\text{FN}}\;F1\;\text{score}=2*\frac{\text{precision}*\text{sensitivity}}{\text{precision}+\text{sensitivity}}$$

Brier score loss, widely employed for quantifying prediction error, captures the disparity between predicted probabilities and observed outcomes. A lower Brier score loss signifies enhanced predictive accuracy of the model. Youden’s J statistic, an inclusive indicator encompassing sensitivity and specificity, provides a holistic assessment of the model’s performance. It ranges from 0 to 1, with higher values denoting superior model performance. All the results of ML models were analyzed using Python version 3.8 (Python Software Foundation, Wilmington, DE, USA).

## Supplemental information

Data S1. Model principles and specific configurations related to STAR Methods (1) Detailed explanation of model principles, related to STAR Methods. (2) The specific model configurations, related to STAR Methods.

Data S2. STROCSS guideline
