# Supplementary material for: Enhancing malignant transformation predictions in oral potentially malignant disorders: A novel machine learning framework using real-world data
Source: iScience. 2025 Feb 18;28(3):112062. doi: 10.1016/j.isci.2025.112062 (PMC11915171; doi:10.1016/j.isci.2025.112062)
Supplement: Data S1. Model principles and specific configurations related to STAR Methods — (1) Detailed explanation of model principles, related to STAR Methods. (2) The specific model configurations, related to STAR Methods. [file mmc1.pdf]

iScience, Volume *28*

Supplemental information

Enhancing malignant transformation predictions in oral potentially malignant disorders: A novel machine learning framework using real-world data

Jing Wen Li, Meng Jing Zhang, Ya Fang Zhou, John Adeoye, Jing Ya Jane Pu, Peter Thomson, Colman Patrick McGrath, Dian Zhang, and Li Wu Zheng

Data S1

1. Detailed explanation of model principles, related to STAR Methods.

Self-attention is a modeling framework first proposed by the Google team[1]. This approach is capable of reducing the dependence on external information and more efficiently capturing the internal relevance of data or features, particularly excelling in modeling long-distance dependencies[2]. The self-attention weights are obtained by first applying linear transformations to the query (Q) and key (K) vectors, followed by calculating the similarity between the transformed vectors. The similarity scores are then normalized using the softmax function to yield the attention weights. Finally, the attention outputs are computed as the weighted sum of the value (V) vectors, based on the obtained attention weights. The core formula underpinning the self-attention mechanism can be expressed as follows:

$$\mathrm{Attention}(Q, K, V) = \mathrm{softmax}\left(\frac{QK^T}{\sqrt{d_k}}\right)V \qquad (1)$$

Where $\sqrt{d_k}$ denotes the scaling factor that controls the size of the dot product, *Q* stands for query; *K* stands for key; and *V* stands for value.

Self-attention mainly calculates the value of attention between elements within a sequence, and is mainly used to obtain the correlation between elements within a sequence, it can correlate the link between any two elements in a sequence by the same calculation step, which is equivalent to shortening the distance between distantly dependent features, so that these features can be utilized more effectively.

The self-attention mechanism primarily focuses on calculating the attention values between elements within a given sequence. This approach is particularly adept at capturing the correlations between elements, as it can establish links between any two elements in the sequence through a unified calculation step. This is akin to shortening the distance between distantly dependent features, thereby enabling these features to be more effectively leveraged by the model. At the core of the self-attention framework is the computation of the correlation between elements in the input sequence. By applying the same calculation step, self-attention can bridge the gap between any two elements, regardless of their relative positions. This ability to model long-range dependencies allows the self-attention mechanism to exploit such interdependent features more efficiently.

The structure of Self-Attention-ANN is shown in Fig. 1B, which contains three main modules: input layer, hidden layer and output layer.

(1) Input layer

The input layer receives the feature vector $x = [x_1, x_2, \ldots, x_n]$, where n is the number of input features. Feature extraction is done on the input data using Self-attention as shown in equation (1).

(2) Hidden layers

Suppose Self-Attention-ANN has L hidden layers and each hidden layer can be represented by the following equation:

For layer l ($l \in \{1,2,\ldots,L\}$), the output $\mathrm{h}^{(l)}$of the hidden layer is obtained from the output $\mathrm{h}^{(l-1)}$ of the previous layer by a linear transformation and an activation function:

$$\mathrm{h}^{(l)} = \mathrm{ReLU}(W^{(l)}\mathrm{h}^{(l-1)} + b^{(l)})$$

where $W^{(l)}$ is the weight matrix of layer l and $b^{(l)}$ is the bias vector of layer l. For layer 1, the input is the original input feature $\mathrm{h}^{(0)} = x$.

(3) Output layer

Assuming that the output of the output layer is y, the value of the probability of the risk of malignant transformation of the OPMDs, then:

$$y = \sigma(W^{(L+1)}h^{(L)} + b^{(L+1)})$$

where $W^{(L+1)}$ is the weight matrix of the output layer, $b^{(L+1)}$ is the bias vector of the output layer, and σ is the Sigmoid activation function.

(4) Loss function

Binary Cross Entropy Loss (BCELoss) was used to compute the loss between the predicted value $\tilde{y}$ and the true label y_{true}. The loss was computed using the binary cross entropy loss (BCELoss):

$$\mathcal{L}(\tilde{y}, y_{true}) = -\frac{1}{B}\sum_{i=1}^{B}[y_{true,i}log(\tilde{y_i}) + (1 - y_{true,i})log(1 - \tilde{y_i})]$$

Reference

[1] Ashish Vaswani, Noam Shazeer, Niki Parmar, Jakob Uszkoreit, Llion Jones, Aidan N. Gomez, Łukasz Kaiser, and Illia Polosukhin. 2017. Attention is all you need. In Proceedings of the 31st International Conference on Neural Information Processing Systems (NIPS'17). Curran Associates Inc., Red Hook, NY, USA, 6000–6010.

[2] Lei, Y., Li, S., Liu, Z., Wan, F., Tian, T., Li, S., Zhao, D., & Zeng, J. (2021). A deep-learning framework for multi-level peptide-protein interaction prediction. Nature communications, 12(1), 5465. https://doi.org/10.1038/s41467-021-25772-4

2. The specific model configurations, related to STAR Methods.

- **Random Forest (RF)**: Gini index as the impurity measure, with 100 trees in the forest.

- **DeepSurv**: The number of nodes for the input layer, first hidden layer, second hidden layer, and output layer were set to 31, 25, 25, and 1, respectively. ReLU activation function was used for all layers, with a learning rate of 0.01, dropout ratio of 0.1, and 400 training epochs.

- **Artificial Neural Network (ANN)**: Input layer with 31 nodes, one hidden layer with 3 nodes, and an output layer with 1 node. ReLU activation function in the hidden layer and sigmoid activation in the output layer. Batch size of 20, 200 epochs, and a learning rate of 0.05 using the Adam optimizer.

- **Self-attention-ANN (SA-ANN)**: Input layer with 31 nodes, three hidden layers with 10 nodes each, and an output layer with 1 node. ReLU activation in the hidden layers and sigmoid activation in the output layer. Batch size of 40, 200 epochs, learning rate of 0.01, dropout ratio of 0.0005, and L2 regularization factor of 0.0001. The Adam optimizer was used.
